# KuSL2023: A standard for Kurdish sign language detection and classification using hand tracking and machine learning

**DOI:** 10.1016/j.mex.2025.103374

**Published:** 2025-05-16

**Authors:** Karwan M. Hama Rawf

**Affiliations:** Department of Computer Science, College of Science, University of Halabja, Halabja, Kurdistan Region, F.R., Iraq

**Keywords:** KuSL2023: A CNN-Based Recognition Method for Kurdish Sign Language Detection and Classification, Sign Language Recognition (SLR), Kurdish Alphabet, Kurdish Sign Language (KuSL), Gesture Recognition, Hand Shape Classification

## Abstract

Sign Language Recognition (SLR) plays a vital role in enhancing communication for the deaf and hearing-impaired communities, yet there has been a lack of resources for Kurdish Sign Language (KuSL). To address this, a comprehensive standard for KuSL detection and classification has been introduced. This standard includes the creation of a real-time KuSL recognition dataset, focusing on hand shape classification, composed of 71,400 images derived from merging and refining two key datasets: ASL and ArSL2018. The ArSL2018 dataset, aligned with the Kurdish script, contributed 54,049 images, while the ASL dataset added 78,000 RGB images, representing 34 Kurdish sign categories and capturing a variety of lighting conditions, angles, and backgrounds. Various machine learning models were employed to evaluate system performance. The CNN model achieved an accuracy of 98.22 %, while traditional classifiers such as KNN and LightGBM reached 95.98 % and 96.94 %, respectively, with considerably faster training times. These findings underscore the robustness of the KuSL dataset, which not only delivers high accuracy and efficiency but also sets a new benchmark for advancing Kurdish Sign Language recognition and broader gesture recognition technology.

• Provides the first standardized dataset for Kurdish Sign Language recognition using 71,400 annotated images.
• Demonstrates high classification accuracy using CNN (98.22 %) and traditional models like KNN and LightGBM.
• Enables real-time hand sign recognition and supports the development of assistive technologies for the deaf community.

## Graphical abstract

**Figure:**
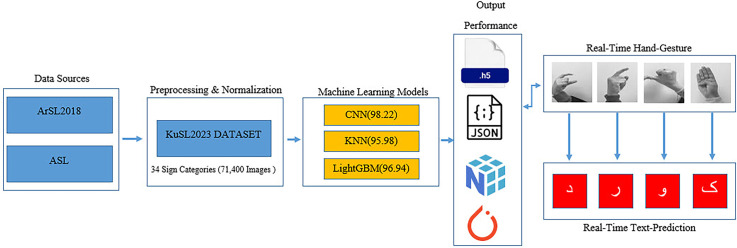

Specifications table

**Table:** 

Subject area:	Computer Science
More specific subject area:	Deep Learning for Sign Language Recognition using Computer Vision and Pattern Analysis
Name of your method:	KuSL2023: A CNN-Based Recognition Method for Kurdish Sign Language Detection and Classification
Name and reference of original method:	Original Method: Convolutional Neural Network (CNN) Reference: Rawf, K.M., A.O. Abdulrahman, and A.A. Mohammed, Improved Recognition of Kurdish Sign Language Using Modified CNN. Computers, 2024. 13(2): p. 37.
Resource availability:	Repository name: Data mendeley (Kurdish Sign Language(KuSL2023)Dataset) Data identification number: (10.17632/6gfrvzfh69.3) Direct URL to data: https://data.mendeley.com/datasets/6gfrvzfh69/2 Instructions for accessing these data: Rawf, Karwan Mahdi; Abdulrahman, Ayub; Ali, Aree (2024), “Kurdish Sign Language(KuSL2023)Dataset”, Mendeley Data, V2, doi: 10.17632/6gfrvzfh69.2 [2]

## Background

Sign language is essential for communication among the deaf and hearing-impaired, but learning it requires significant time and effort. Advances in computer vision and deep learning offer a solution through automated systems that translate sign language into text or speech. Effective systems depend on high-quality, diverse datasets. The KuSL2023 dataset addresses this need by offering a comprehensive collection of Kurdish Sign Language (KSL) images captured under various lighting conditions and backgrounds, including indoor, outdoor, bright, dark, and natural lighting. This variety ensures models trained on KuSL2023 perform well in real-world scenarios [1]. Unlike other publicly available datasets, which often lack gesture variability and are collected under uniform conditions (e.g., [[3], [4], [5], [6], [7], [8]] (KuSL2023 includes a range of scenarios involving different positions and heights, making it a valuable tool for developing and testing robust sign language recognition systems. By bridging gaps in existing resources, KuSL2023 enhances communication accessibility for Kurdish-speaking communities and supports advancements in machine learning and computer vision.

## Method details

KuSL2023 is an establishing Kurdish Sign Language (KSL) dataset for gesture detection and machine learning research. It helps create real-world solutions, notably hearing-impaired assistive devices, establishes a standard for model evaluation, and provides practical instruction [1], Principal contributions of proposed dataset:

• Unique Dataset for Kurdish Sign Language: The KuSL2023 dataset is a pioneering resource specifically designed for Kurdish Sign Language (KSL). It enables the development of automated sign language recognition systems tailored for Kurdish users, advancing research in gesture recognition and machine learning within this linguistic context.
• Cross-Domain Applications: The dataset holds potential for various domains including gesture recognition, machine learning, and natural language processing. Researchers can leverage KuSL2023 for training and testing models across different applications, enhancing the adaptability and performance of systems in real-world scenarios.
• Enhancing Model Performance: By incorporating KuSL2023 with existing datasets, researchers can improve model robustness and adaptability. The dataset helps in expanding sample sizes, varying conditions, and reducing biases, which is crucial for developing more accurate and generalizable sign language recognition models.
• Educational and Developmental Use: The dataset serves as an educational resource for students and practitioners in fields such as signal processing, machine learning, and computer vision. It provides a practical tool for learning and applying advanced techniques in gesture recognition and sign language technology.
• Benchmarking and Validation: KuSL2023 offers a valuable benchmark for evaluating and comparing different sign language recognition models. Researchers can use it to test algorithms, validate performance, and develop new methods for improving recognition accuracy and reliability in diverse conditions.
• Real-World Application Potential: The dataset's comprehensive collection of KSL signs under varied conditions supports the development of real-world applications such as communication tools and assistive technologies for individuals with hearing impairments. It facilitates the creation of applications that bridge communication gaps between Kurdish sign language users and the broader community.

The proposed dataset is assessed using various models, achieving significant accuracy compared to other datasets. It shows high efficiency and delivers crucial results without additional accuracy-enhancing techniques. Details are discussed in the “ Method Validation” section.

The KuSL2023 dataset focuses on static hand poses for recognizing Kurdish Sign Language (KuSL), comprising 34 signs representing letters of the Kurdish alphabet. Like many sign language recognition systems, KuSL2023 distinguishes between static and dynamic gestures, prioritizing static, single-hand gestures for its dataset creation, a method also seen in other sign language systems such as Bengali Sign Language (BdSL), which categorizes signs into static and dynamic types [[9], [10], [11], [12]]. The use of static signs allows for a structured representation of the Kurdish alphabet, similar to other sign languages that employ static hand poses for specific alphabets [9,10].

KuSL2023 presents a comprehensive collection of 71,400 images, ensuring a diverse set of examples with varied lighting and background conditions, allowing for robust model training. This diversity makes the dataset particularly useful for real-world applications, addressing a limitation often seen in sign language datasets, where uniform conditions are common. Unlike the imbalance issues faced by other datasets that require additional effort to balance [13], In KuSL2023, while the number of grayscale images varies across classes, the RGB images are uniformly distributed, with each class containing an equal number of images and consistent image sizes. This approach ensures that the RGB images contribute to balanced model training while the grayscale images, despite counts, maintain a roughly similar size across classes.

The dataset consists of images of hand gestures performed by volunteers, captured under varying conditions similar to the approach used in other sign language datasets, such as BdSL. In contrast to systems that incorporate speaker-specific factors like age and gender, KuSL2023 focuses solely on gesture variation and environmental diversity, making it an excellent resource for training models on visual feature extraction.

Faculty members from the University of Halabja’s Computer Science Department ensured high-quality data collection by adhering to established guidelines, aligning with best practices seen [9], where participants were guided in performing hand signs [14,15]. While there is no inclusion of speaker-specific variations, the diversity in hand gestures and environmental conditions improves the dataset’s applicability for machine learning models, similar to how other sign language datasets have incorporated environmental variability to enhance accuracy [16]. Moreover, KuSL2023 is designed to support assistive technologies, providing a foundation for translating hand signs into text and voice, a growing area of interest in sign language recognition systems globally. Its utility extends beyond traditional sign language applications, opening doors for further research in the field of visual pattern recognition and sign language-based communication technologies.

The Kurdish alphabet consists of 26 consonants and 8 vowels, which is comparable to many other languages such as English (26), French (26), Greek (24), Arabic (28), and Russian (33). The main vowels in the Kurdish alphabet are , while the remaining characters represent consonants. The KuSL2023 dataset is meticulously organized into a main directory titled “Kurdish Sign Language 2023 (KuSL2023),” containing 34 subdirectories, each representing a specific letter of the Kurdish alphabet, including . Each subdirectory contains between 1,290 and 3,000 images in both RGB and grayscale formats, with the images systematically named to ensure organized structure. Notably, KuSL2023 incorporates RGB colour images for specific Kurdish letters, including , while grayscale images represent the remaining letters. This combination of RGB and grayscale formats enhances the dataset's visual diversity, and grayscale images represent the remaining letters, with each RGB class containing 3,000 images and the grayscale classes starting from approximately 1,290 images. The disparity in the number of grayscale images arises from a refinement process, wherein images displaying near-identical hand positions or formatting were removed to improve class representation. This careful balancing minimizes redundancy while preserving essential gesture variations, aligning with best practices in dataset creation.

**Fig. 1:**
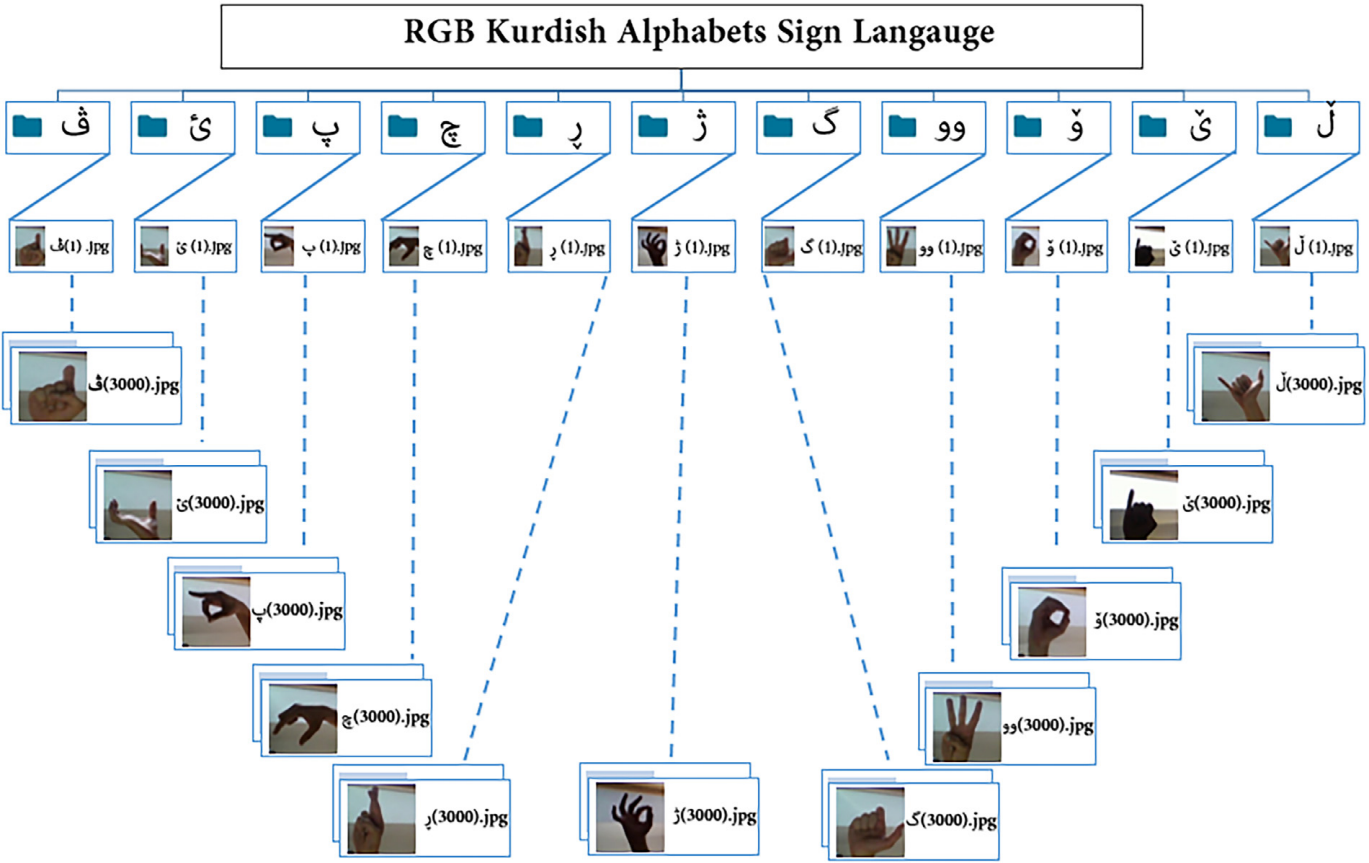

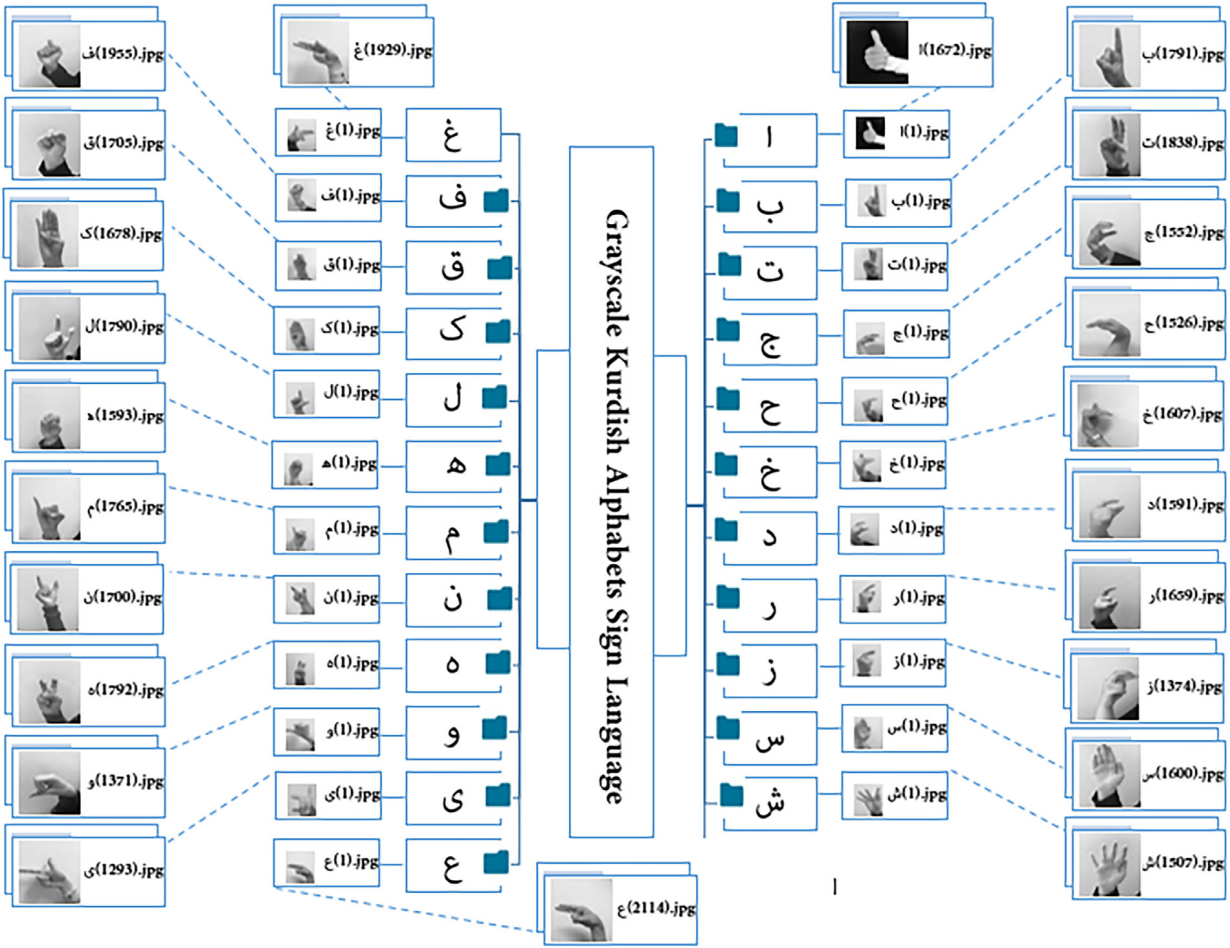
(a): Structure of the proposed dataset: RGB Kurdish Alphabets in Sign Language. (b): Structure of the proposed dataset: Grayscale Kurdish Alphabets in Sign Language.

The KuSL2023 dataset comprises a total of 71,400 images, offering a comprehensive resource for training and evaluating sign language recognition models. These images are categorized into two sections: the first section includes 33,000 RGB images across 11 classes, while the second section consists of 38,400 grayscale images spanning 23 classes. Each class contains between 1,290 and 3,000 images, with the alphabetic signs classified based on the shape and orientation of hand signals. The varied lighting conditions and environmental diversity of the images mirror real-world scenarios, enhancing the dataset’s practical utility. The dataset is organized around 34 Kurdish letters, as shown in Fig. 1, which provides a visual illustration of the RGB and grayscale images for the respective Kurdish letters. Table 1 details the number of images available for each class. The dataset is also divided into two parts: a detection section containing full-frame images and a recognition section focusing solely on the hand signs. Samples from the dataset are attached to the repository link.

**Table 1:** The Image distribution among classes in the KuSL2023 dataset.




Alif*, Ha*= appears only in non-initial positions in Kurdish words

The structure offers a rich and diverse dataset for machine learning research in sign language recognition, particularly for Kurdish Sign Language, and stands out as a valuable resource for developing assistive technologies aimed at translating hand signs into text or voice. Currently, there is no adequate dataset available for automated Kurdish sign identification, which presents a significant limitation in the field. This research seeks to overcome this challenge by developing a dataset specifically for Kurdish Sign Language (KuSL) recognition. Spoken by over 40 million people across regions in Turkey, Iraq, Iran, and Syria, the Kurdish language includes three main dialects: Sorani, Badini, and Hawrami. Individual voice tone is a key identifier in biometric systems, and improving communication accessibility through Dialect Recognition Systems (DRS) and KuSL recognition is vital [17,18].

Preliminary experiments determined that an average of over 2,000 images per class provides sufficient variation for effectively recognizing each Kurdish sign. Various machine learning algorithms were trained on differing sample sizes and evaluated on a separate test set. While the sample size may be considered relatively small, especially for the grayscale images, where each class is approximately 3 MB, the size of each image file is reduced to around 10 KB. This reduction ensures efficient storage while maintaining diversity. The dataset was deemed adequate for this study, with efforts made to ensure it captures the full range of Kurdish Sign Language by sourcing data from multiple origins. Table 1 visually illustrates the distribution of RGB and grayscale images across different Kurdish letters, detailing the number of images per class and emphasizing the balance between RGB and grayscale formats.

## Method validation

The experimental design of the KuSL2023 dataset focuses on providing a publicly available Kurdish Sign Language alphabet set, including both consonants and vowels, with variations in environmental and lighting conditions. The dataset is structured in a standard format, allowing users to pre-process and apply it to train or test supervised, semi-supervised, and unsupervised machine learning and deep learning models [5,17,19,20]. Additionally, it aims to encourage further research into Kurdish Sign Language translation. The main goal of this study is to assess the performance of various machine learning models in real-time recognition of Kurdish Sign Language using the KuSL2023 dataset. The experimental framework involves training and evaluating multiple algorithms to determine their effectiveness in classifying and predicting sign language gestures. The dataset is divided into training, validation, and test sets to ensure a thorough performance evaluation and mitigate overfitting, simulating real-world conditions to test the models' robustness across diverse scenarios. In terms of materials, the KuSL2023 dataset comprises 71,400 images representing 34 distinct Kurdish letters captured in both RGB and grayscale formats. High-resolution images were selected to ensure clarity and consistency. The images feature a variety of backgrounds, including solid and multi-coloured, to enhance the dataset's robustness. Each image focuses exclusively on the hand performing the sign, with other objects deliberately excluded. These images were taken under varied lighting conditions, including both indoor and natural light, to reflect real-time environments. Additionally, images were captured from multiple angles and postures to ensure comprehensive data representation.

The images were collected using high-quality smartphones to capture diverse lighting conditions and hand postures, enhancing the dataset's robustness. Pre-processing steps included standardizing image resolution and normalizing pixel values. RGB images, typically larger in size (approximately 3 MB per class), were complemented by grayscale images with significantly smaller file sizes (below 10 KB per image) to ensure effective dataset management and adequate class representation. Several machine learning models were employed, including Convolutional Neural Networks (CNNs) like TensorFlow and Keras, and traditional classifiers like KNN and LightGBM chosen for their effectiveness in image classification.

Quantization and pruning techniques were implemented to reduce model size and improve real-time performance. Although data augmentation was not applied during the initial dataset collection, it is planned for future experiments to enhance model robustness further. The machine learning models were evaluated using accuracy, precision, recall, and F1-score metrics to assess their effectiveness in real-time sign language recognition. The details of the models used to evaluate the dataset are illustrated in Table 2. The objective is to achieve substantial results that will advance the field of sign language recognition and offer practical solutions for applications benefiting the deaf community and other sectors where sign language is pivotal.

**Table 2:** Classification results for traditional and ensemble machine learning classifiers on the KuSL2023 Dataset.

Classifier	Accuracy ( %)	Precision ( %)	Recall ( %)	F1 Score ( %)	Time (Seconds)
CNN_ TensorFlow and Keras	98.22 %	97.79 %	97.73 %	97.75 %	6679
KNN	95.98 %	96.02 %	95.98 %	95.99 %	79.58
Logistic Regression (LR)	85.46 %	85.50 %	85.46 %	85.45 %	269.77
Light-GBM	96.94 %	96.20 %	96.15 %	96.16 %	481.37

To achieve a balanced representation between RGB and grayscale images in the KuSL2023 dataset, each RGB-based class was standardized to contain 3,000 images. This decision reflects the fact that RGB images encode more complex visual features due to their multi-channel nature, which generally requires a larger volume of data for effective feature extraction and model generalization. In contrast, grayscale images, which contain fewer visual cues, were curated to include between 1,290 and 3,000 samples per class. A manual refinement process was applied to eliminate near-duplicate grayscale samples, ensuring intra-class diversity. During model training, stratified sampling techniques were employed to mitigate the impact of class size variation, thereby maintaining equitable learning across both RGB and grayscale image modalities.

To compute the scores for each sign individually, the values of True Positive (TP), True Negative (TN), False Positive (FP), and False Negative (FN) cases were extracted for every sign. The performance metrics—Accuracy, Precision, Recall, and F1 Score (F-measure)—were then assessed using the following equations:

(1) $$Accuracy=\frac{TP+TN\;}{TP+TN+FP+FN}$$

(2) $$Precision\;\left(PPV\right)\;=\frac{TP}{TP+FP}$$

(3) $$Sensitivity\;or\;Recall\;\left(TPR\right)=\frac{TP}{TP+FN}$$

(4) $$F1=\frac{2TP\;}{2TP+FP+FN}$$

**Fig. 2:**
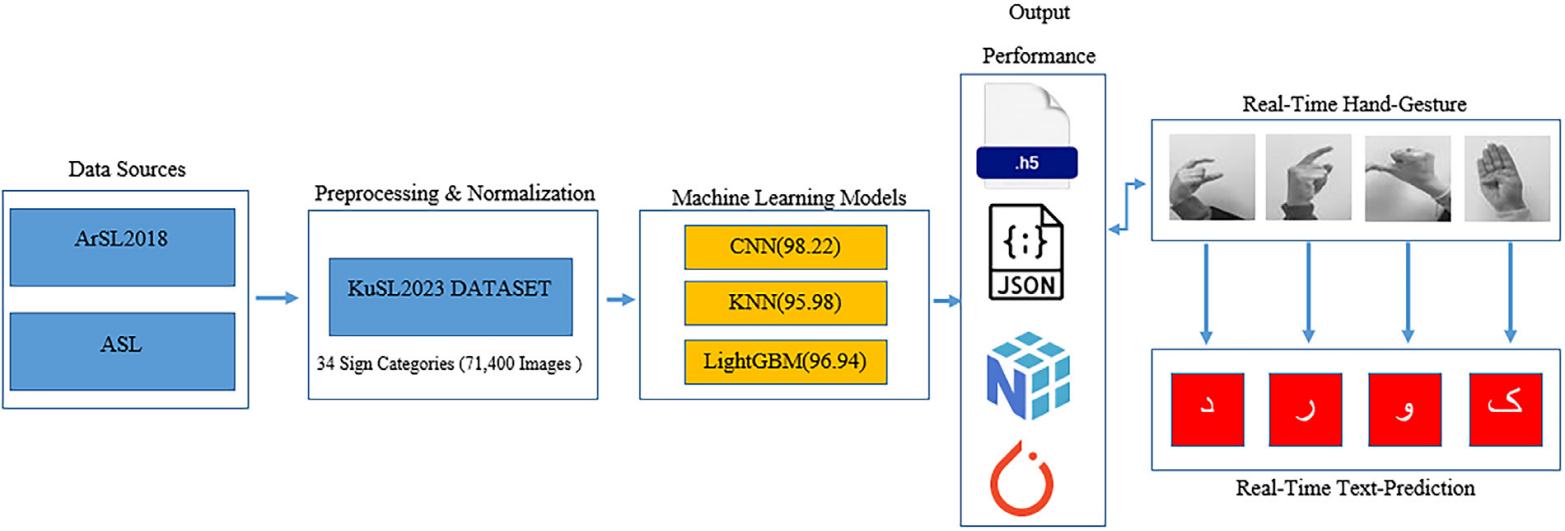
Flow diagram of the KuSL2023 dataset pipeline from data collection to real-time sign language recognition.

The complete process of dataset construction, preprocessing, model training, and real-time application for KuSL2023 is summarized in the flow diagram shown in Fig. 2.

A flow diagram has been included to illustrate the full KuSL2023 data pipeline, capturing the process from data acquisition to application. The pipeline begins with the integration of two key datasets—ASL (78,000 RGB images) and ArSL2018 (54,049 grayscale images)—as the primary sources for Kurdish sign representation. These images undergo preprocessing, including hand tracking and normalization, to prepare them for model training. The curated images are then organized into the KuSL2023 dataset, consisting of 71,400 labeled samples spanning 34 Kurdish sign categories in both RGB and grayscale formats. Subsequently, classification models such as CNN, KNN, and LightGBM are trained and evaluated on the dataset. The outputs of this pipeline include .h5 files for trained models, .npy for processed data arrays, .pt for model weights, and .json for storing evaluation metrics. The pipeline concludes with the deployment of these models for real-time applications, demonstrating their potential for use in assistive technologies that support communication for the deaf and hearing-impaired communities. Following the completion of the data pipeline, the performance outcomes of the CNN, KNN, and LightGBM classifiers on the KuSL2023 dataset are summarized in Table 2.

In the evaluation of the KuSL2023 dataset, various classifiers were employed to assess the dataset's potential for Kurdish Sign Language recognition. The CNN model, utilizing TensorFlow and Keras, achieved impressive metrics with an accuracy of 98.22 %, precision of 97.79 %, recall of 97.73 %, and an F1 score of 97.75 %, although approximately 6679 seconds were required for training. In comparison, traditional classifiers like KNN and LightGBM demonstrated strong performance, with accuracies of 95.98 % and 96.94 %, respectively, and notably reduced training times of 79.58 seconds and 481.37 seconds. These results were obtained without implementing any techniques to enhance performance and were based on initial training iterations, underscoring the dataset's robustness. The metrics indicate that the KuSL2023 dataset is not only capable of delivering high accuracy but also efficient training times across various models, demonstrating its value as a critical resource for advancing sign language technology.

Real-time testing of the KuSL2023 dataset illustrates its efficiency, as shown in Table 3, which presents results for specific signs. This method not only demonstrates the dataset's capability for accurate gesture recognition but also highlights its potential for real-world applications, such as communication aids for the hearing impaired. Assessing performance in real-time allows researchers to identify improvement areas, enhancing the dataset's utility in sign language recognition systems.

**Table 3:** Performance metrics for various sign language recognition attempts across multiple trials.

The real-time prediction results for the Kurdish Sign Language (KuSL2023) dataset, as shown in Table 3, reveal varying accuracy levels: 80 % for the signs ج and گ, and 90 % for  and ڕ. Notably, the model incorrectly predicted ج as د and ز, while ح was misclassified as خ and ڕ as ت. Additionally, the sign گ was mistakenly identified as ڤ and ا. These outcomes underscore the model's adaptability in real-time scenarios and its potential for practical application. Future improvements in accuracy could be achieved by integrating advanced techniques such as data augmentation, transfer learning, or ensemble methods, which would enhance the model's performance and robustness in recognizing a broader range of gestures.

To ensure transparency and reproducibility, the pseudocode presented in Box 1 provides a detailed representation of the complete workflow used to construct, preprocess, and evaluate the KuSL2023 dataset. This step-by-step outline reflects the real implementation logic applied across all models—starting from the integration of ASL and ArSL2018.

Box 1 **KuSL2023 Preprocessing and Evaluation Pipeline.**

# KuSL2023 Preprocessing and Model Evaluation Pipeline

# Step 1: Load Datasets

load ASL_dataset    # RGB images for 11 Kurdish letters

load ArSL2018_dataset   # Grayscale images for 23 Kurdish letters

# Step 2: Filter and Align Kurdish Alphabet with Sign Representations

for letter in Kurdish_alphabet:

 if letter has similar shape in ASL: select 3000 RGB images (unaltered)

 elif letter is present in ArSL2018:

  filter grayscale images for uniqueness

  resize images to standard size (64 × 64)

  store in grayscale class folder

# Step 3: Dataset Construction and Balancing

for class in dataset:

 if class uses RGB: ensure exactly 3000 images

 elif class uses grayscale: retain between 1290–3000 refined images

# Step 4: Dataset Preprocessing Based on Model Type

# For KNN and LightGBM (Grayscale only)

for each image:

 convert to grayscale

 resize to (64 × 64)

 flatten to 1D vector

 normalize pixel values

 append image and label

apply LabelEncoder to convert class names to numeric labels

split dataset into train/test using stratified sampling (80/20)

scale features using StandardScaler

# For CNN (RGB)

use tf.keras.image_dataset_from_directory() with:

ȃimage_size = (64 × 64)

 batch_size = 64

 validation_split = 0.1

 color_mode = 'rgb'

apply:

- Rescaling to [0,1]

- CNN architecture (Conv2D, MaxPool, Dropout, Dense)

- Compile with Adam optimizer and softmax activation

# Step 5: Model Training and Evaluation

for model in [CNN, KNN, LightGBM]:

 train on training data

 validate on validation set (if applicable)

 predict on test set

 evaluate using: (Accuracy, Precision (macro/weighted), Recall, F1 Score, Runtime tracking)

# Step 6: Visualization (CNN only)

 - Plot training/validation accuracy and loss

 - Compute and visualize confusion matrix

Alt-text: Unlabelled box

To support reproducibility and methodological transparency, the structured pseudocode in Box 1 illustrates the full pipeline used to build, preprocess, and evaluate the KuSL2023 dataset. The process begins with the integration of two primary sources—ASL for RGB images and ArSL2018 for grayscale images—mapped to the Kurdish alphabet based on visual similarity. Signs visually aligned with ASL are directly represented with 3,000 RGB images per class, while those resembling Arabic-script signs are derived from ArSL2018, undergoing refinement to remove near-duplicates and resizing for uniformity. Class balancing ensures 3,000 samples for each RGB class and between 1,290 to 3,000 images for grayscale classes. For grayscale-based models (KNN and LightGBM), all images are converted to 64 × 64, flattened, normalized, and scaled; label encoding and stratified train-test splits are also applied. For the CNN model, the RGB data is loaded using a TensorFlow pipeline, with images resized to 64 × 64 and normalized. The classifiers—CNN, KNN, and LightGBM—are then trained and evaluated using standard performance metrics, including accuracy, precision, recall, F1 score, and runtime. This pseudocode offers a replicable and practical framework for researchers working in gesture recognition and sign language classification.

### Data collection

Technological advancements have significantly transformed the process of identifying specific signs, particularly in sign language recognition. With machine learning, deep learning, and artificial intelligence, researchers can now analyse and detect sign patterns more effectively. These innovations are crucial in preserving linguistic diversity, safeguarding cultural heritage, and facilitating cross-cultural communication by improving the interpretation of sign language patterns.

In developing the Kurdish Sign Language (KuSL2023) dataset, the initial focus was gathering high-quality samples accurately representing Kurdish sign language. Various electronic resources were utilized, alongside collaborations with disability organizations and media sources such as TV programs, interviews, news segments, and documentaries. The collected data came in diverse formats, including videos, images, and historical paintings, later converted into image files for segmentation; for each sign representing a letter in the Kurdish alphabet, over 1,200 images were captured, resulting in 71,400 images. These images depict gestures from both hands in varying positions and contexts. The dataset is structured into 34 directories, corresponding to each letter of the Kurdish alphabet, with each directory containing an average of 2,000 JPG image files. The images were captured from several adults, each with unique appearances, ensuring diversity. Strict protocols were followed during the data collection to maintain high data quality, with rigorous recording procedures and an extensive review to detect and resolve inconsistencies. Noise reduction techniques were applied during preparation to ensure clear, high-quality data. The KuSL2023 dataset, comprising 71,400 labelled images across 34 categories, is now publicly available to researchers and can be used to develop automated systems through techniques such as machine learning, computer vision, and deep learning.

### Sound segmentation and pre-processing

To construct the KuSL2023 dataset, each Kurdish letter was first mapped to corresponding signs from the Arabic Sign Language (ArSL2018) and American Sign Language (ASL) datasets Approximately 10 letters were selected from the ASL dataset, each containing 3,000 RGB images with varying backgrounds, positions, and lighting conditions, and these were included directly without additional pre-processing or segmentation. For the ArSL2018 dataset, which closely aligns with the Kurdish script, over 20 folders were selected to match specific Kurdish letters. Nearly duplicated images were removed, and the remaining grayscale images were resized to maintain consistency with the RGB images. This resizing was necessary to accommodate the varying sizes of grayscale images and ensure uniformity for training the machine learning models. In this study, a specialized approach was adopted for the annotation and preprocessing of the KuSL2023 dataset, with a particular focus on image analysis. Instead of relying entirely on automated Python-based methods for labeling and preprocessing, a combination of manual oversight and Python scripts was employed. This approach enabled a more detailed and accurate understanding of the image data, allowing for more precise classification and refinement of annotations. By using this method, the nuances and subtleties of image features were better captured, leading to improved data quality for subsequent machine learning tasks.

### Labelling

The dataset labeling process is vital for creating new datasets, involving the assignment of meaningful and specific labels to data. This process entails categorizing or classifying data based on defined criteria. Labeling involves two key steps for the proposed dataset. First, each class folder is renamed using Kurdish letters rather than English script, with each class separated into individual folders. Next, files within these folders are labelled sequentially. For RGB image files, labels range from Kurdish letter(1) to Kurdish letter(3000), while for grayscale images, labels extend from Kurdish letter(1) to the last image number in the class, due to variations in the number of files per grayscale class as illustrated in Table 1. This approach ensures each file is accurately identified according to its respective class. Python code was used for this labeling process, as it simplifies and speeds up the renaming and updating of class names and files compared to manual methods, particularly when files are deleted.

## Limitations

For the KuSL2023 dataset, limitations in its creation are "Not applicable." Initially, training machine learning models yielded low results, but after extensive tuning and precise dataset partitioning, accuracy improved significantly. The main limitations for the models used are: CNNs can have high computational complexity, leading to longer training times; ResNet, with its deep architecture, can be resource-intensive; EfficientNet offers a balance of accuracy and efficiency but may require considerable computational power; and CNN-LSTM models, though effective for sequential data, can be slow to train due to their combined complexity.

## Ethics statements

The KuSL2023 dataset was developed by integrating two publicly available datasets, originally designed for research purposes, with the goal of enhancing Kurdish Sign Language recognition. This dataset solely comprises hand gesture images, ensuring no personal information of the participants is included. All contributors participated voluntarily and provided informed consent. The data collection involved only standard imaging techniques, with no invasive or clinical testing, thus posing no physical impact on the participants. This research adheres to ethical standards by focusing on non-invasive methods and gesture recognition. Both source datasets are cited as part of the dataset's legacy. The KuSL2023 dataset is open access, supporting further research while respecting privacy and ethical considerations.

## CRediT authorship contribution statement

**Karwan M. Hama Rawf:** Conceptualization, Data curation, Investigation, Methodology, Validation, Visualization, Writing – original draft, Writing – review & editing.

## Declaration of competing interest

The authors declare that they have no known competing financial interests or personal relationships that could have appeared to influence the work reported in this paper.

## Data Availability

The data that has been used is confidential.
